# Mental disorders on admission to jail: A study of prevalence and a comparison with a community sample in the north of France

**DOI:** 10.1192/j.eurpsy.2020.38

**Published:** 2020-04-27

**Authors:** Thomas Fovet, Laurent Plancke, Alina Amariei, Imane Benradia, Fanny Carton, Aminata Sy, Maeva Kyheng, Grégory Tasniere, Ali Amad, Thierry Danel, Pierre Thomas, Jean-Luc Roelandt

**Affiliations:** 1 Univ. Lille, Inserm, U1172—Lille Neuroscience & Cognition—Equipe Plasticity & SubjectivitY, F-59000 Lille, France; 2 CHU Lille, Pôle de Psychiatrie, F-59000 Lille, France; 3 Fédération régionale de recherche en psychiatrie et santé mentale Hauts-de-France (F2RSM Psy), Saint-André-Lez-Lille, France; 4 Univ. Lille, CNRS, UMR 8019 - CLERSE - Centre Lillois d’Études et de Recherches sociologiques et Économiques, F-59000 Lille, France; 5 EPSM Lille-Métropole, Centre Collaborateur de l’Organisation Mondiale de la Santé pour la Recherche et la Formation en Santé Mentale, Lille, France; 6 ECEVE, UMRS 1123, Université Paris Diderot, Sorbonne Paris Cité, INSERM, Paris, France; 7 Univ. Lille, CHU Lille, ULR 2694 - METRICS : évaluation des technologies de santé et des pratiques médicales, F-59000 Lille, France; 8 CHU Lille, Département de Biostatistiques, F-59000 Lille, France; 9 EPSM Lille-Métropole, France

**Keywords:** France, mental health, prevalence, prison

## Abstract

**Background.:**

The aim of the present study was to estimate prevalence rates of psychiatric and substance use disorders in male and female prisoners on admission to prison in the north of France and compare the frequency of these disorders to the general population.

**Methods.:**

This cross-sectional survey on Mental Health in the Prison Population (MHPP), conducted between March 2014 and April 2017, interviewed 653 randomly selected men and women who had recently been committed to the French general population prison system in the Nord and Pas-de-Calais departments. For each subject, the Mini International Neuropsychiatric Interview (MINI), a standardized psychiatric interview, was used to screen for psychiatric and substance use disorders. The prevalence rates were then compared with data from the Mental Health in the General Population (MHGP) survey, a general population survey that used the same assessment methodology as MHPP in the Nord and Pas-de-Calais departments. A control sample was taken from the MHGP survey with a ratio of one case (MHPP) to three controls (MHGP) matching on age and sex.

**Results.:**

The sample was primarily composed of French men, most of them single with low educational levels at the time of imprisonment. The mean age was 31.7 (standard deviation = 9.9; min = 18; max = 67). Most of the subjects included were first-time prisoners. The prevalence of affective disorders among newly incarcerated individuals was 31.2% with higher rates for major depressive disorder (27.2%). The prevalence of anxiety disorders was 44.4% with higher rates for generalized anxiety disorder (25.2%). The prevalence of psychotic syndromes was 6.9%. The prevalence of substance use disorders was 53.5% and a suicide risk was identified in 31.4% of the prisoners interviewed. Higher prevalence rates were found in the MHPP when compared with the MHGP for all psychiatric and substance use disorders assessed except for dysthymia and current isolated psychotic syndrome.

**Conclusions.:**

Our study shows very high levels of prevalence for psychiatric and substance use disorders in recently committed French prisoners.

## Introduction

High levels of psychiatric morbidity are consistently reported in prisoners from many countries [1] and international meta-analyses have found that prisoners in many places have elevated rates of psychiatric disorders compared with the general population for diagnoses such as psychosis, depression, personality disorders, and addictive disorders [2, 3]. However, the previously published meta-analyses are based on studies with several limitations, such as small samples or the use of diagnostic tools that have not always been adapted to a prison environment. In particular, significant variations can be observed between countries due to differences in the functioning of judicial institutions.

Recently, the nongovernmental organization Human Rights Watch severely criticized the management of patients suffering from psychiatric disorders in French prisons, labeling the incarceration and inability to access appropriate care a “double punishment” for these patients [4]. The situation of prisons in France is indeed worrying as the number of prisoners has been constantly increasing since the early 2000s. On June 1, 2019, more than 70,000 people were detained under severely overcrowded conditions (overall occupancy rate of 117%; nearly 1,500 mattresses on the floor). The situation is even more worrying in jails (remand prisons; in France, these are institutions to which sentenced persons with less than 2 years of their prison sentence remaining or accused persons awaiting appearance before a court are sent for detention). Overall, nearly 20,000 prisoners are incarcerated in an institution with an occupancy rate of over 150% (sometimes exceeding 200%).

Considering this particular context, very few studies have focused on the prevalence of mental health disorders in French prisons. The epidemiological study that is currently being used as a reference in France corresponds to the study by Falissard et al. conducted between 2003 and 2004 and recently supplemented by a study in the prison of Ducos, Martinique [5, 6]. This survey involved a random sample of 800 French male prisoners incarcerated in 20 penitentiary facilities (jails and prisons). By the consensus of a team of clinicians (including at least one senior psychiatrist), the diagnoses were established based on DSM-IV criteria. One of the clinicians used a standardized tool: the Mini International Neuropsychiatric Interview (MINI) [5]. The results showed that 36% of inmates have at least one psychiatric illness considered as marked to severe (i.e., a rating of 5, 6, or 7 on the Global Clinical Impressions Scale). Prevalence rates for a diagnosis given independently by both clinicians were: 3.8% for schizophrenia, 17.9% for major depressive disorder, 12.0% for generalized anxiety, and 10.8% for drug dependence. This study recruited 800 subjects regardless of the stage of imprisonment and thus provides information on the point-prevalence of mental disorders among prisoners. However, this methodology may result in an overrepresentation of long-term prisoners in comparison to other types of inmates who are imprisoned over time, emphasizing the long-term effects of the prison environment [7]. To rigorously study the mental health status of the prisoners in France, it is crucial to supplement this study with a survey that includes consecutively admitted prisoners (i.e., an admission study). This type of survey can identify the social environment prior to admission [7] and the criminal policies of a country.

Only a few admission studies have been conducted in the last 20 years in metropolitan France [8–13]. Furthermore, the interpretation of their results remains very limited because they either did not use a standardized diagnostic tool [8–12] or were conducted on limited samples with a high participation refusal rate [13]. As such, the aim of the present study was to establish prevalence rates of mental disorders in male and female prisoners on admission to jail in the north of France and to compare the frequency of these disorders to the general population. To this end, the Mental Health in the Prison Population (MHPP) survey was conducted in two French departments (Nord and Pas-de-Calais). The results were compared with a community sample from the same geographic area with matching sex and age range.

## Methods

### Mental Health in the Prison Population survey

The cross-sectional MHPP survey conducted between March 2014 and April 2017 by the Fédération Régionale de Recherche en Psychiatrie et Santé Mentale (Regional Federation for Research in Psychiatry and Mental Health, F2RSM Psy) and the World Health Organization Collaborating Center in mental health (WHOCC Lille) interviewed 653 randomly selected men and women who had recently been committed to the French general population prison system in Nord and Pas-de-Calais (departments in the north of France).

### Sample

The number of subjects to be included was statistically calculated based on the Clopper–Pearson method [14]. The lowest expected prevalence for a psychiatric disorder evaluated by the MINI was for psychotic syndrome with an estimated prevalence rate of 5%. In these conditions, the number of individuals to be recruited was 655.

Subjects were randomly approached for inclusion from admission lists of consecutively committed people in eight of the nine remand prisons of Nord and Pas-de-Calais in France: *Arras*, *Douai*, *Dunkerque*, *Sequedin*, *Annoeullin*, *Longuenesse*, *Maubeuge*, and *Valenciennes.* One remand prison (*Béthune*) refused to be included in the study. The number of recently incarcerated subjects (male and female) per year was collected for each facility (based on data provided by the prison administration for the year 2010) and the number of men and women to be recruited per center was prorated. Subjects were included in the study if they met the following criteria: (a) provided informed consent to participate in the survey, (b) spoke French, (c) were aged 18 years or older, (d) were free of any mental or psychological incapacity to participate due to acute decompensation of a psychiatric disorder or severe substance withdrawal (i.e., health condition requiring emergency hospitalization), and (e) had been incarcerated for less than 72 h (sentenced or on remand). For each remand prison, the recruitment days were selected at random. Sundays and public holidays were not eligible days. During a recruitment day, all committed people were consecutively recruited. When people rejected participation, the next person on the list was approached until the number of participants expected for each center was recruited. People with a safeguarding vulnerable adult legal measure were included.

Exclusion criteria were the inability to communicate in the French language, a lack of capacity to provide informed consent and mental or psychological incapacity to participate due to acute decompensation of a psychiatric disorder or severe substance withdrawal.

Ethical approval was obtained via the “Comité de Protection des Personnes” (CPP) (IDRCB 2012 A0144835), the “Commission Nationale Informatique et Liberté” (CNIL) (MMS/VCS/AR152838), and the “Agence nationale de sécurité du médicament et des produits de santé” (ANSM) (130500B-31). All interviewees provided written informed consent. The data was collected between March 2014 and April 2017. Under strict conditions of confidentiality, each participant was interviewed for 45–60 min within the prison medical unit.

### Sociodemographic characteristics

Sociodemographic data (age, gender, marital and employment status, monthly income, educational level, housing, previous imprisonment, and religious practice) was gathered for each subject using a structured questionnaire. Age was categorized into four age groups (18–23 years old, 24–29 years old, 30–38 years old, and over 39 years old). Income was categorized as low (<€840/household per month), medium (€840–2,520/household per month), or high (>€2,520/household per month).

### Assessment of psychiatric symptoms

For each subject, the MINI (French version 5.0.0), a standardized psychiatric interview, was used to screen for psychiatric disorders as defined by the 10th International Classification of Diseases (ICD-10) [15]. In comparison to the Composite International Diagnostic Interview (CIDI), the MINI has good to very good kappa values. All of the MHPP interviewers (nurses and psychologists) were trained to conduct the MINI over a 1-day session provided by WHOCC experts.

The following psychiatric disorders were assessed using the MINI: manic episodes (F30), depressive disorders (current and recurrent, F32 and F33), current dysthymia (F34.1), alcohol use disorders [i.e., dependence (F10.2) and abuse (F10.1)], substance use disorders [i.e., substance abuse (F1(x).1) and substance dependence (F1(x).2)], psychotic syndromes [isolated or recurrent, F2(x); always confirmed by a senior psychiatrist or psychologist] and anxiety disorders, that is, panic disorder with or without agoraphobia (F41.0 and F40.01), social anxiety disorder (SAD, F40.1), generalized anxiety disorder (GAD, F41.1), and post-traumatic stress disorder (PTSD, F43.1). Suicide risk was also screened.

### Statistical analyses

#### Characteristics of the sample

The statistical analyses were run using SAS 9.4 (SAS Institute Inc., Cary, NC). Sociodemographic characteristics and prevalence rates of mental disorders were calculated as percentage values with a confidence interval (CI) of 95%. Age was calculated as a mean and standard deviation from the mean.

#### Comparison with the general population

In order to compare the prevalence of psychiatric disorders in the MHPP sample with the prevalence of the same disorders in the general population, we used regional data from the Mental Health in the General Population (MHGP) survey [16–20]. The cross-sectional MHGP survey conducted by the WHOCC interviewed 12,568 subjects in Nord and Pas-de-Calais, France, between 2001 and 2008. Subjects were selected from 14 sites (900 subjects per site) using a quota-sampling method. This method provides a sample of subjects with a sociodemographic profile representative of the general population in terms of age, sex, educational level, and occupational category according to census figures from 1999 provided by the *French National Institute of Statistics and Economic Studies* (INSEE). Subjects were included in the study if they met the following criteria: (a) provided informed consent to participate in the survey, (b) spoke French, (c) were aged 18 years or older, (d) were residing in Nord or Pas-de-Calais, and (e) were neither institutionalized nor homeless. MHGP and MHPP used the same methodology to assess psychiatric symptoms (assessment with the MINI).

A control sample was taken from the MHGP survey with a ratio of one case (MHPP) to three controls (MHGP) matching on age and sex. To ensure full comparability between the two samples, all people not residing in the Nord and Pas-de-Calais departments (*n* = 31) were excluded from the MHPP sample for this analysis. Case and control disorder prevalence was compared by logistic regression with an odds ratio (OR) and 95% CI calculation between the two groups for each disorder studied.

#### Analysis of psychiatric and substance use comorbidities

To study the frequency of multiple diagnoses and comorbidity, we compared the proportion of patients without any psychiatric or substance use disorder to those with 1–4 (or more) psychiatric or substance use disorder(s) as identified with the MINI (Chi-square test).

#### Analysis of sociodemographic covariates

Chi-square tests were used to detect significant relationships between the sociodemographic characteristics and presentation of at least one psychiatric disorder. Covariates with *p*-values less than 0.2 in bivariate analysis were included in a multivariate logistic regression model to examine their impact on the probability of presenting at least one of the following psychiatric disorders: depressive disorders (current and recurrent; F32 and F33); current dysthymia (F34.1); manic episodes (F30); agoraphobia (F40.0); panic disorders (F41.0); SAD (F40.1); GAD (F41.1); PTSD (F43.1); psychotic syndromes isolated or recurrent (F2(x)); substance abuse (F1(x).1); and substance dependence (F1(x).2).

## Results

### Recruitment

A total of 934 subjects were approached but only 655 were eligible and provided consent to participate to the study (participation rate: 70.1%). Two participants were excluded because of incomplete data. In total, 653 participants were interviewed.

For the current study, 31 people did not reside in the Nord and Pas-de-Calais departments and were therefore excluded. The data of 622 participants were used in the final analysis.

### Sociodemographic characteristics

Sociodemographic data was gathered for each subject. This data is reported in Table 1. The majority of the sample was composed of French men, most of them single and with low educational levels at the time of imprisonment. The mean age was 31.7 (standard deviation = 9.9; min = 18; max = 67). Most of the subjects included were first-time prisoners.

**Table 1.** Sociodemographic characteristics of the sample (*n* = 622).

	*N*	%
Age band		
18–23 years old	145	23.3
24–29 years old	164	26.4
30–38 years old	167	26.8
39+ years old	146	23.5
Gender		
Male	599	96.3
Female	23	3.7
Education level		
No education/primary level	299	48.1
Secondary level	313	50.3
University level	9	1.4
No response	1	0.2
Monthly income level		
Low (<€840/household)	304	48.9
Medium (€841–2,520/household)	269	43.2
High (>€2,520/household)	41	6.6
No response	8	1.3
Marital status		
Married or co-residing with partner	270	43.4
Single	272	43.7
Separated or divorced	78	12.5
Widowed	1	0.2
No response	1	0.2
Previous imprisonment		
Yes	261	42.0
No	360	57.8
No response	1	0.2
Employment		
Yes	177	28.5
No	445	71.5
Permanent home		
Yes	554	89.1
No	68	10.9
Safeguarding vulnerable adult legal measure		
Yes	12	1.9
No	610	98.1
Religious practice		
Practicing	141	22.7
Believer but not practicing	196	31.5
Believer with no indication	45	7.2
Non-believer	230	37.0
No response	10	1.6

### Mental health and substance use disorders

The prevalence of psychiatric and substance use disorders is reported in Table 2. The prevalence of affective disorders among newly incarcerated individuals was 31.2% with higher rates for major depressive disorder (27.2%). The prevalence of anxiety disorders was 44.4% with higher rates for generalized anxiety disorder (25.2%). The prevalence of psychotic syndromes was 6.9%. The prevalence of substance use disorders was 53.5% and a suicide risk was identified in 31.4% of the prisoners interviewed.

**Table 2.** Comparison between prevalence rates of psychiatric and substance use disorders among recently admitted prisoners (MHPP) and the general population (MHGP) in the Nord and Pas-de-Calais departments of France.

	MHPP (*n* = 622)		MHGP (*n* = 1,866)		Comparison		
	Prevalence (%)	CI 95%	Prevalence (%)	CI 95%	Odds Ratio	CI 95%	*p*-value
Any affective disorder	31.2	27.5–34.8	14.6	13.0–16.2	2.7	2.1–3.3	<0.001
*Major depression*	27.2	23.7–30.7	11.3	9.9–12.7	2.9	2.3–3.7	<0.001
*Recurrent major depression*	15.8	12.9–18.6	5.1	4.1–6.1	3.4	2.6–4.6	<0.001
*Dysthymia*	3.2	1.8–4.6	2.1	1.4–2.7	1.6	0.9–2.7	0.113
*Past or current manic episode*	7.1	5.1–9.1	2.7	2.0–3.5	2.7	1.8–4.1	<0.001
Any anxiety disorder	44.4	40.5–48.3	21.2	19.4–23.1	3.0	2.4–3.6	<0.001
*Agoraphobia*	6.4	4.5–8.4	1.9	1.3–2.6	3.5	2.2–5.5	<0.001
*Panic disorder*	13.0	10.4–15.7	3.6	2.8–4.5	4.0	2.8–5.5	<0.001
*Panic disorder with agoraphobia*	1.1	0.3–2.0	0.4	0.1–0.7	3.0	1.1–8.7	0.039
*Social anxiety disorder*	6.4	4.5–8.4	3.6	2.8–4.5	1.8	1.2–2.7	0.004
*Generalized anxiety disorder*	25.2	21.8–28.7	12.9	11.4–14.4	2.3	1.8–2.9	<0.001
*Post-traumatic stress disorder*	5.0	3.3–6.7	0.9	0.4–1.3	6.1	3.3–11.2	<0.001
Any substance use disorders	53.5	49.6–57.5	12.9	11.4–14.4	7.8	6.3–9.6	<0.001
*Alcohol abuse*	11.7	9.2–14.3	2.6	1.9–3.4	4.9	3.4–7.2	<0.001
*Alcohol dependence*	23.5	20.1–26.8	5.5	4.5–6.6	5.3	4.0–6.9	<0.001
*Drug (excluding nicotine) abuse*	4.5	2.9–6.1	0.2	0.0–0.4	27.7	14.1–111.2	<0.001
*Drug (excluding nicotine) dependence*	26.7	23.2–30.2	6.7	5.6–7.8	6.1	4.8–7.8	<0.001
Heroin^a^	9.2	1.9-16.4	0.2	0.0–1.3	46.9	16.9–129.9	<0.001
Cocaine/crack^a^	9.3	2.9-15.7	1.1	0.0–3.4	9.1	5.4–15.0	<0.001
Marijuana^a^	45.2	40.4-50.0	6.9	4.4–9.4	11.2	8.8–14.2	<0.001
Amphetamines^a^	1.1	0.0-6.4	0.4	0.0–3.6	2.6	0.9–7.3	0.061
Psychotropic drugs^a^	1.3	0.0–7.2	0.3	0.0–3.2	4.0	1.4–11.7	<0.001
Opioid substitution therapy^a^	2.6	0–9.6	0.2	0.0–2.2	12.3	4.1–36.9	<0.001
Any psychotic disorder	6.9	4.9–8.9	2.3	1.6–3.0	3.1	2.0–4.9	<0.001
*Current isolated psychotic syndrome*	0.5	0.0–1.0	0.2	0.0–0.4	2.3	0.5–10.2	0.283
*Past isolated psychotic syndrome*	1.6	0.6–2.6	0.4	0.1–0.7	3.8	1.5–9.7	0.005
*Current recurrent psychotic syndrome*	2.7	1.5–4.0	1.1	0.6–1.6	2.5	1.3–4.7	0.006
*Past recurrent psychotic syndrome*	2.1	1.0–3.2	0.5	0.2–0.9	4.0	1.7–9.2	0.001
Suicide risk	31.4	27.7–35.0	12.5	11.0–14.0	2.9	2.3–3.5	<0.001
*Low*	15.8	12.9–18.6	8.4	7.1–9.6	2.1	1.6–2.7	<0.001
*Moderate*	3.4	2.0–4.8	2.5	1.8–3.2	1.4	0.8–2.3	0.257
*High*	12.2	9.6–14.8	1.7	1.1–2.2	8.2	5.4–12.6	<0.001

A ratio of one case (MHPP) to three controls (MHGP) matching on age and sex was used.

Abbreviations: MHGP, Mental Health in the General Population survey; MHPP, Mental Health in the Prison Population survey.

a Use, abuse, or dependence.

### Comparison with the general population

Prevalence was higher in the MHPP than in the MHGP for major depressive disorder (OR = 2.9, CI = 2.3–3.7), generalized anxiety disorder (OR = 2.3, CI = 1.8–2.9) and psychotic syndrome (OR = 3.1, OR = 2.0–4.9). Higher prevalence rates were found in the MHPP when compared with the MHGP for all the psychiatric and substance use disorders assessed except for dysthymia and current isolated psychotic syndrome (see Table 2 for details of all psychiatric and substance use disorders).

### Analysis of psychiatric and substance use comorbidities

To explore the weight of co-occurrence of psychiatric and substance use disorders, we compared the proportion of patients without any psychiatric or substance use disorders and patients with one or more psychiatric or substance use disorder(s) in the MHGP and MHPP (see Table 3). The vast majority of the MHGP sample (66.5%) had no psychiatric or substance use disorders, whereas most of the newly incarcerated people of the MHPP (63.3%) had at least one psychiatric or substance use disorder, with 41.6% of the sample presenting two or more diagnoses.

**Table 3.** Number of mental disorders identified with the MINI in the MHGP (*n* = 1866) and MHPP (*n* = 622).

Number of mental disorders	MHPP (*n* = 622)		MHGP (*n* = 1866)	
0	228	36.7	1,241	66.5
1	135	21.7	387	20.8
2	116	18.6	148	7.9
3	63	10.1	58	3.1
4+	80	12.9	32	1.7
Total	622	100.0	1,866	100.0

Abbreviations: MHGP, Mental Health in the General Population survey; MHPP, Mental Health in the Prison Population survey.

### Analysis of sociodemographic covariates

The bivariate analysis revealed that all covariates, except religious practice, were associated with significant prevalence differences where at least one psychiatric or substance use disorder (*p* < 0.05) was present. However, only the effects of the MHGP/MHPP group, employment, and marital status remained significant in the logistic multivariate regression analysis (see Supplementary Material). Thus, for the whole sample (MHGP and MHPP), entering prison (OR = 2.50), divorce/separation (OR = 2.44), and unemployment (OR = 1.67) were associated with a higher risk of having at least one psychiatric or substance use disorder.

## Discussion

In this paper, we presented the first results of the MHPP survey. Three main findings emerged. First, at close to two thirds of the total sample population of newly incarcerated people, the prevalence of psychiatric and substance use disorders was high. Second, for most of the psychiatric disorders assessed, this prevalence was significantly higher than a sample of the general population matched for age and sex and living in the same geographic area. Third, comorbidities were very frequent among recently incarcerated prisoners as 41.6% of the sample had two or more psychiatric or substance use disorders identified with the MINI.

While the international literature documents a high prevalence of psychiatric disorders in prisoners, French studies in this field are scarce. The results obtained in the MHPP study show higher prevalence rates than previous studies in the field (particularly those found by Falissard et al. in their 2006 study) [5]. In our study, 63.3% of the prisoners presented at least one disorder while Falissard et al. found that 33.9% of their sample suffered from at least one disorder identified by the MINI. Prevalence rates of mood disorders were quite similar (31.2% vs. 28.6% in Falissard’s study) but anxiety disorders were much more frequent in our sample (44.4% vs. 24.0% in Falissard’s study) as well as substance use disorders (53.5% vs. 14.1% in Falissard’s study). The prevalence of psychotic syndromes identified in our study was much lower than that found in Falissard’s work (6.9% vs. 17.3%). These differences could be explained by the two methodological designs used (assessment at intake vs. assessment during incarceration), resulting in two different populations of interest: new arrivals versus long-time prisoners. By assessing the disorders on arrival, our study may have overestimated anxiety disorders among inmates. Indeed, upon entering detention, people have to cope with various environmental stressors, such as deprivation of liberty, bullying, and breakdown of family ties [21]. Similarly, by assessing the disorders during detention, Falissard et al. may have underestimated substance use disorders as imprisonment may hinder the use of drugs and alcohol (even if it does not necessarily preclude it). Our results are in line with recent meta-analysis showing that approximately one quarter of newly incarcerated prisoners of both sexes had an alcohol use disorder, and that the prevalence of a drug use disorder was at least as high in men, and higher in women [22]. With regard to psychotic syndromes, the validity of the MINI in a population of prisoners has already been questioned, particularly because derealization, ideas of persecution and even delusions have to be carefully considered in the very particular context of prison [23]. As a result, it should be hypothesized that psychotic syndromes have been overestimated to a greater extent in long-time prisoners interviewed in Falissard’s study than in our sample of newly incarcerated people. Other possible explanations include that people with psychotic disorders could be given longer sentences or that imprisonment and isolation could be involved in the occurrence of psychotic symptoms [24], which would lead to an over-representation in Falissard’s study compared to our study.

Our results are also largely consistent with previous prison admission studies conducted in high-income Western countries. For example, Trestman et al. demonstrated that more than two of three inmates met the criteria for at least one lifetime psychiatric disorder, almost half for an anxiety disorder, and more than one-third for an affective disorder in the United States [25]. In a study by Gunter et al., screening 320 men and women entering the Iowa prison system, more than 90% of newly committed offenders in the Iowa prison system met criteria for a current or lifetime psychiatric disorder. The most frequent were substance use disorders (90%) and mood disorders (54%) [26]. As in our study highlighting the very high proportion of people with several mental or substance use disorders who are incarcerated in France (41.6% of our sample had two or more diagnoses), Gunter et al. found that two-thirds of offenders had three or more disorders identified with the MINI-plus [26]. In Dutch prisons, Bulten et al. found a prevalence of 18% for current depression and 8% for life-time psychosis diagnosis. Including substance abuse, 57% of the participants presented at least one disorder identified with the MINI and a suicide risk was identified for 35% of prisoners [27]. Studies in low- and middle-income countries are rare but a recent study in Chile identified drug and/or alcohol use disorders in the year prior to admission in 76%, current major depression in 54% and current non-affective psychotic disorders in 8% of male prisoners [7].

For all the psychiatric and substance use disorders assessed, except for dysthymia and current isolated psychotic syndrome, the prevalence rates were significantly higher in our sample than in a general population sample from the same geographic area matched on age and sex (see Table 2). The difference between MHPP and MHGP samples was particularly significant for substance use disorders (OR estimated at 7.8 for any substance use disorder). The OR was measured at 2.7 for affective disorders, 3.0 for anxiety disorders (with an OR estimated at 6.1 for PTSD), and 3.1 for psychotic syndromes. A high suicide risk was estimated to be 8.2 times more frequent in our sample than in the general population sample. This result is consistent with previous studies showing very high suicide rates in France (more than 100 suicides per 100,000 prisoners) when compared with other high-income countries [28, 29]. These findings should raise concerns about the adequacy of current screening program for psychiatric and substance use disorders in French prisons and most importantly, the mental health care system for prisoners. Despite some changes in recent years, the quality of mental health care varies significantly between prisons in France [30]. Only 26 (out of 188) benefit from a Service medico-psychologique regional (France’s regional medical-psychological service for correctional settings; SMPR) that includes a team of psychiatrists, nurses and psychologists and a number of day hospital beds. In many prisons, there is a severe shortage of mental health professionals, which means that access to primary mental health care is not provided for all prisoners. The creation of the Unités hospitalières spécialement aménagées (specially equipped hospital units; UHSA) which are full-time inpatient wards for inmates [31–33], in 2010, has only allowed a moderate improvement of this situation, particularly because there are only nine UHSA (with a total capacity of 440 beds) for the 70,000 French prisoners.

This study has several limitations. First, it only recruited recently committed prisoners in the Nord and Pas-de-Calais departments. As a result, caution is needed before generalizing the results for the entire population of France. Second, women only represented 3.5% of our total sample (*n* = 23), which considerably limits the interpretation and generalizability of the results for this population. The study is not statistically powerful enough to detect significant differences between the male and female participants, whereas significant differences in the mental health status of incarcerated men and women remain well documented [3, 34]. Third, some limitations from the clinical assessment method should be noted. Because we wanted to assess the prevalence of substance use and psychiatric disorders on admission, the assessment interview was conducted within the first 72 h of incarceration. As a result, the levels of distress, which are highest shortly after admission, may have caused an overestimation of some disorders. In addition, the diagnoses are based on the MINI and no medical records were available. The validity of the MINI in a population of prisoners has already been questioned [23]; however, several recently published prison mental health studies used the MINI [35, 36] and it has been validated as a suitable screening tool in prison settings [37]. It is also important to note that no assessment of personality disorders has been conducted. Furthermore, a limitation for the comparison of the prevalence data between the prison population and the general population arises from a time lag of about 10 years between the two studies. The prevalence data in the general population might have changed over time but no more recent data is available. Finally, no information about the penal situation of the interviewees was available in this study.

## Conclusion

Our study shows very high levels of prevalence for psychiatric and substance use disorders in recently committed French prisoners. The detection of these disorders on admission is essential in order to initiate adequate treatment. However, these results also emphasize the need to initiate primary prevention programs aimed at reducing social deprivation or substance use disorders, and to develop diversion programs or alternatives to imprisonment for people with severe mental disorders in France.

## Data Availability

The data on which this manuscript is based are not publicly available. However, data from the MHPP are available upon request, under supervision of the principal investigator (PI) of the study. Access to data is subject to a formal data sharing agreement in order to ensure that any other researcher accessing the data follows appropriate ethical standards. The PI of the MHPP (Pr. Pierre Thomas) can be contacted (pierre.thomas@chru-lille.fr) at all times to request data: researchers can submit a research plan, describing its background, research questions, variables to be used in the analyses and an outline of the analyses. If such a request is approved, a written agreement will be signed stating that the data will only be used for addressing the agreed research questions, and not for other purposes.

## Supplementary material

For supplementary material accompanying this paper visit http://dx.doi.org/10.1192/j.eurpsy.2020.38. click here to view supplementary material
