# Supplementary material for: Mental disorders on admission to jail: A study of prevalence and a comparison with a community sample in the north of France
Source: Eur Psychiatry. 2020 Apr 27;63(1):e43. doi: 10.1192/j.eurpsy.2020.38 (PMC7355167; doi:10.1192/j.eurpsy.2020.38)
Supplement: Supplementary file 1 [file S0924933820000383sup001.docx]

| **Supplementary table. Factors associated with psychiatric disorders in the whole sample (MHGP and MHPP)** | | | | | | | | | | | | | | |
| --- | --- | --- | --- | --- | --- | --- | --- | --- | --- | --- | --- | --- | --- | --- |
|  |  |  | **Bivariate analysis** | | | | | |  | **Logistic regression** | | | |  |
| Having at least 1 disorder |  |  | Yes |  | No |  | Total |  |  | (having at least 1 disorder) | | | |  |
|  |  |  | N | *%* | N | *%* | N | P Khi2 |  | OR | CI95% |  | P^1^ | p^2^ |
|  | *Total* |  | *1,019* | *41.0* | *1,469* | *59.0* | *2,488* |  |  |  | *-* |  | *-* |  |
| Group | MHGP |  | 625 | *33.5* | 1,241 | *66.5* | 1,866 | <0.0001 |  | Ref. | - |  | - | <0.001 |
|  | **MHPP** |  | **394** | ***63.3*** | **228** | ***36.7*** | **622** |  |  | **2.50** | **1.99-3.14** |  | **<0.001** |  |
| Marital status (MV=17) | Single |  | 457 | *41.3* | 650 | *58.7* | 1,107 | <0.0001 |  | Ref. | - |  | - | <0.001 |
|  | Married or co-residing with partner |  | 421 | *36.5* | 734 | *63.5* | 1,155 |  |  | 0.98 | 0.81-1.19 |  | 0.854 |  |
|  | **Separated or divorced** |  | **124** | ***64.2*** | **69** | ***35.8*** | **193** |  |  | **2.44** | **1.73-3.48** |  | **<0.001** |  |
|  | Widowed |  | 7 | *43.8* | 9 | *56.3* | 16 |  |  | 1.30 | 0.47-3.62 |  | 0.613 |  |
| Education level (MV=4) | No education / primary level |  | 495 | *40.6* | 724 | *59.4* | 1,219 | <0.0001 |  | 1.05 | 0.82-1.35 |  | 0.683 | 0.130 |
|  | Secondary level |  | 368 | *46.9* | 416 | *53.1* | 784 |  |  | 1.26 | 0.96-1.64 |  | 0.091 |  |
|  | University level |  | 155 | *32.2* | 326 | *67.8* | 481 |  |  | Ref. | - |  | - |  |
| Employment | Yes |  | 482 | *32.7* | 993 | *67.3* | 1,475 | <0.0001 |  | Ref. | - |  | - | <0.001 |
|  | **No** |  | **537** | ***53.0*** | **476** | ***47.0*** | **1,013** |  |  | **1.67** | **1.36-2.05** |  | **<0.001** |  |
| Monthly income level (MV=59) | Low |  | 301 | *58.1* | 217 | *41.9* | 518 | <0.0001 |  | 1.34 | 0.98-1.84 |  | 0.065 | 0.168 |
|  | Medium |  | 554 | *38.0* | 902 | *62.0* | 1.456 |  |  | 1.11 | 0.87-1.41 |  | 0.382 |  |
|  | High |  | 141 | *31.0* | 314 | *69.0* | 455 |  |  | Ref. | - |  | - |  |
| Religious practice | Believer but not practising |  | 337 | *44.0* | 429 | *56.0* | 766 | 0.069 |  | 1.16 | 0.94-1.43 |  | 0.173 | 0.416 |
|  | Practising |  | 150 | *42.5* | 203 | 57.5 | 353 |  |  | 0.91 | 0.70-1.20 |  | 0.514 |  |
|  | Believer with no indication |  | 144 | *35.5* | 262 | 64.5 | 406 |  |  | 1.01 | 0.78-1.31 |  | 0.940 |  |
|  | Non-believers |  | 360 | *40.5* | 529 | 59.5 | 889 |  |  | Ref. | - |  | - |  |
|  | Missing values |  | 28 | *37.8* | 46 | 62.2 | 74 |  |  | 1.25 | 0.73-2.14 |  | 0.415 |  |

OR = Odds ratio - CI = Confidence interval - P^1^ = modality - p^2^ = variable – MV = Missing value.
